# Epigenetics in Precision Nutrition

**DOI:** 10.3390/jpm12040533

**Published:** 2022-03-28

**Authors:** Xiang Li, Lu Qi

**Affiliations:** 1 Department of Epidemiology, School of Public Health and Tropical Medicine, Tulane University, New Orleans, LA 70112, USA; xli23@tulane.edu; 2 Department of Nutrition, Harvard T.H. Chan School of Public Health, Boston, MA 02115, USA

**Keywords:** precision nutrition, epigenomics, DNA methylation, type 2 diabetes, obesity, CVD

## Abstract

Precision nutrition is an emerging area of nutrition research, with primary focus on the individual variability in response to dietary and lifestyle factors, which are mainly determined by an individual’s intrinsic variations, such as those in genome, epigenome, and gut microbiome. The current research on precision nutrition is heavily focused on genome and gut microbiome, while epigenome (DNA methylation, non-coding RNAs, and histone modification) is largely neglected. The epigenome acts as the interface between the human genome and environmental stressors, including diets and lifestyle. Increasing evidence has suggested that epigenetic modifications, particularly DNA methylation, may determine the individual variability in metabolic health and response to dietary and lifestyle factors and, therefore, hold great promise in discovering novel markers for precision nutrition and potential targets for precision interventions. This review summarized recent studies on DNA methylation with obesity, diabetes, and cardiovascular disease, with more emphasis put in the relations of DNA methylation with nutrition and diet/lifestyle interventions. We also briefly reviewed other epigenetic events, such as non-coding RNAs, in relation to human health and nutrition, and discussed the potential role of epigenetics in the precision nutrition research.

## 1. Introduction

Nutrition is among the most important factors affecting human health, and healthy diet intervention has become a mainstream approach in the prevention and treatment of human diseases, such as obesity, type 2 diabetes (T2D) and cardiovascular disease (CVD). The inter-individual variability in response to dietary intervention has long been noted [1]. It is now generally accepted that the “one-size-fits-all” dietary intervention strategy does not achieve the best result for complex disease prevention and treatment. Therefore, new dietary intervention strategies, considering the inter-individual difference, merit urgent attention. In recent years, ‘precision nutrition’ has emerged as a new area of nutrition research, with a particular focus on revealing the individual variability in response to diets, which is determined by a variety of intrinsic factors, including genomic, epigenomic, and microbiomic variations, among others. The advances in high-performance technologies provide the opportunity to discover the role of various omics data in the pathophysiology and management of complex diseases [2]. Such rich and personalized information, along with access to the big data of electronic health records and rapidly evolving computational power and bioinformatic methods, are paving the way for precision nutrition [2,3].

Currently, precision nutrition research is mainly focused on the genome and gut microbiome, while the epigenome is largely uncharted. Epigenomic modifications include the hereditable and reversible changes in gene function that occur without changing the DNA sequence, such as DNA methylation, histone modification, and non-coding RNA regulation. Among them, DNA methylation has been the most-studied epigenetic event so far. DNA methylation is a covalent chemical modification of DNA, in which the hydrogen H5 of cytosine is replaced by a methyl group, under the catalyzation by DNA methyltransferases (DNMTs). DNA methylation regulates gene expression, either by impeding the binding of transcriptional proteins or by recruiting proteins that are involved in gene repression. DNA methylation may provide a mechanistic link between environmental factors and gene expression and, therefore, potential targets for diseases prevention, through modifications of environmental factors, such as diets [4,5,6].

To date, epigenome-wide association studies (EWASs) have identified various DNA methylation markers, associated with various diseases [7], and emerging studies further explored the role of DNA methylation in affecting the response to diet and lifestyle modifications. In this review, we briefly summarized the recent discoveries on DNA methylation with cardiometabolic diseases, including obesity, T2D, and CVD. We particularly reviewed the studies on the relations between nutrition and DNA methylation, followed by presenting some recent works on DNA methylation in response to weight loss and other lifestyle interventions. This review also included studies related to other epigenetic events, such as microRNA (miRNA) and long non-coding RNA (lncRNA). In addition, we discussed the potential role of epigenetics in precision nutrition research.

## 2. DNA Methylation in Obesity, T2D, and CVD

### 2.1. DNA Methylation and Obesity

The global prevalence of obesity nearly tripled between 1975 and 2016 [8]. It is expected that more than 1 billion adults will be affected by obesity by 2025, if the current trend continues [8]. The alarming increase in obesity is paralleled with the profound shift from the “traditional” to “obesogenic” environments, such as the abundance of energy-dense food and reduced physical activity. Obesity is a multifactorial disease, resulting from environmental factors, genetic variations, and their interactions. Emerging data have also implicated epigenetic modifications as a potential player.

The first large-scale EWAS of the association between adult body mass index (BMI) and DNA methylation was conducted by Dick et al., in 2014 [9]. The study included 459 European individuals from the Cardiogenics Consortium for the discovery cohort. Primary and secondary replications were conducted among 339 unrelated northern European participants from the MARseille THrombosis Association (MARTHA) cohort and 1789 white European participants from the Kooperative Gesundheitsforschung in der Region Augsburg (KORA) cohort. Whole-blood DNA methylation was measured by the Infinium HumanMethylation450 array. Five CpG sites across three different genes were found to be associated with BMI. Among them, three CpG sites, namely cg22891070, cg27146050, and cg16672562, located in intron 1 of hypoxia-inducible factor 3a (*HIF3A*), were successfully replicated in MARTHA and KORA. A year later, Demerath et al. conducted a cross-ethnic investigation on DNA methylation (leukocyte DNA samples assayed by the Illumina HumanMethylation450 array) with obesity among 2097 African American adults in the Atherosclerosis Risk in Communities (ARIC) study [10]. Replications were conducted among 2377 White adults in the Framingham Heart Study (FHS), using whole-blood DNA and 991 White participants in the Genetics of Lipids Lowering Drugs and Diet Network (GOLDN) Study, using CD4+ T cell DNA, followed by cross-tissue association analysis among 648 women in the Multiple Tissue Human Expression Resource (MuTHER) cohort, using adipose tissue DNA. A total of 76 CpG sites were recorded from the discovery phase among the African American participants and 37 of them were successfully replicated in FHS and GOLDN, including CpG sites near *CPT1A*, which has been previously reported to be associated with BMI by Aslibekyan et al. [11], and CpG sites near *ABCG1*. Moreover, a total of 18 CpG sites were associated with BMI in the adipose tissue, including markers near the previously identified *CPT1A* and *ABCG1* loci, and a number of novel adiposity-related loci, such as *LYS6GE*, *KDM2B*, *RALB*, *PRRL5*, *LGALS3BP*, *C7orf50*, *PBX1*, *EPB49* and *BBS2*. Of note, the *HIF3A* probes, previously reported by Dick et al. [9], were confirmed in the discovery phase in African American participants, but not replicated in FHS and GOLDN. Later on, Mendelson et al. conducted an association study of BMI and whole blood DNA methylation (assayed by Illumina HumanMethylation450 array) from 3743 participants FHS and Lothian Birth Cohorts (LBC), with independent replication in three independent cohorts of 4055 participants [12]. The study identified 83 CpG sites, robustly associated with BMI across cohorts. Mendelian randomization was also used to infer the potential causal association between BMI and differential methylation. A substantial proportion of the differentially methylated sites (16/83) were found to be secondary to BMI, rather than the opposite. In contrast, one of the 83 CpG sites, cg11024682 at the *SREBF1* locus, demonstrated evidence of a causal effect on BMI. However, the cross-sectional nature of the study limited the definitive causal determination [12]. Another important study was conducted by Wahl et al., in which a total of 187 CpG sites were found to be significantly associated with BMI [13]. One of the identified CpG sites, cg26663590 (*NFATC2IP*), was shown to be causally associated with BMI by Mendelian randomization. Such association was confirmed by the followed analysis in longitudinal analyses. Of note, the study also found that a Methylation Risk Score (MRS), calculated by summing up the methylation values at each of the markers associated with T2D and weighted by marker-specific effect size, could predict future risk of T2D, independent of traditional T2D risk factors, such as BMI, waist-to-hip ratio, glucose, and HbA1c. In addition, this study supported the view that DNA methylation was a consequence of BMI. A recent EWAS, performed among 1192 17-year-old adolescents from the Raine Study, found that DNA methylation at *RalGDS/AF-6*, *RAPH1*, *MSI2*, and *SLC25A10* was associated with concurrent BMI and waist circumference [14]. In addition, DNA methylation at *RAPH1*, *MSI2*, and *SLC25A10* was also found to be associated with some early life factors, such as maternal smoking, pre- and early pregnancy BMI, and gestional weight gain, suggesting that these could be potential robust markers across lifecourse. It is worth noting that previous EWASs were predominantly performed in European ancestries. A recent cross-sectional analysis identified 116 CpG sites associated with BMI and 8 CpG sites with waist circumference among an Asian popuplation, with moderate consistency compared with other ethnic groups, indicating potential unique DNA methylation markers in the Asian population [15]. A brief summary of the selected DNA methylation markers associated with obesity is shown in Table 1.

### 2.2. DNA Methylation and T2D

The prevalence of T2D is paralleled with that of obesity, which is a major risk factor for T2D [16,17]. It is estimated that by 2030, 7079 individuals per 100,000 will have T2D [18,19]. Except for obesity [20], many other environmental factors are associated with T2D, such as poor diet [21,22], physical inactivity [23], unhealthy sleeping behaviors, etc. [24,25]. However, the underlying pathophysiology mechanisms linking many of these factors with T2D onset and progression are not well understood. There is growing interest in exploring the role of epigenetic mechanisms, particularly DNA methylation, in linking the environmental factors to T2D [26].

So far, many CpG sites have been discovered to be associated with prevalent T2D, although in relatively modest sample sizes (Table 1) [27,28,29,30,31,32]. For example, Dayeh et al. analyzed the DNA methylation of more than 450,000 CpG sites in pancreatic islets from 15 T2D patients and 34 non-diabetic controls [33]. They found a total of 1649 CpG sites annotated to 843 genes, including *TCF7L2*, *FTO*, and *KCNQ1*, differentially methylated in T2D islets. In addition, 102 of the differentially methylated genes, including *CDKN1A*, *PDE7B*, *SEPT9* and *EXOC3L2*, exhibited differential gene expressions in the T2D islets. Previous studies also reported DNA methylation in peripheral blood, which offers easier assessment due to its noninvasive nature. Florath et al. conducted an EWAS among 1515 German older adults [27]. Genome-wide methylation in whole blood was measured by the Illumina Infinium HumanMethylation450 BeadChip. At the discovery stage, a total of 39 CpG sites were significantly associated with prevalent T2D, after adjustment for multiple comparison, and one CpG site, cg19693031 located within the *TXNIP* gene, remained significantly associated with T2D at replication stage. A lower level of methylation at cg19693031 was associated with increasing fasting glucose and HbA1c concentration. A recent large meta-EWAS of prevalent T2D in a European population, using peripheral blood DNA methylation, confirmed three previously identified CpG sites at *TXNIP*, *ABCG1*, and *CPT1A*, and also reported three novel CpG sites associated with prevalent T2D among Europeans, namely cg00144180 (*HDAC4*), cg16765088 (near *SYNM*) and cg24704287 (near *MIR23A*) [34]. Besides, analysis of differentially methylated regions (DMR) based on the meta-analysis results was also conducted in this study, and 77 T2D-assocaited DMRs were identified. Furthermore, additional analysis in a diabetic-free population, the Avon Longitudinal Study of Parents and Children (ALSPAC) cohort, revealed that most of the six sites from this meta-analysis were associated with categories of glucose tolerance, age, sex, white-cell types, and other clinical phenotypes. A recent study also reported that *VDR* genes are differently methylated between T2D patients and controls [35] Moreover, adipose tissue from discordant twin pairs revealed 5 CpG sites annotated to gene promoters of differentially expressed miRNAs were hypermethylated among T2D patients compared to controls [36].

Evidence from EWASs in prospective cohorts also reported differentially methylated CpG sites associated with T2D incidence [29,37,38]. For example, Chambers et al. conducted a large, prospective, nested case-control study of DNA methylation, among 25,375 Indian Asian and European participants, in the London Life Sciences Prospective Population (LOLIPOP) study [38]. Epigenome-wide DNA methylation was assessed by the Illumina HumanMethylation450 array. The study identified an association between differential methylation at five genetic loci (*ABCG1*, *PHOSPHO1*, *SOCS3*, *SREBF1*, and *TXNIP*) and risk of future T2D incidence among Indian Asians and Europeans. In addition, an MRS combining the five CpG sites was associated with incident T2D, independent of established T2D risk factors (relative risk quartile 1 vs. quartile 1: 3.51, 95% CI: 2.79–4.42, *p* = 1.3 × 10^−26^). Another prospective study was performed among 1264 participants from the EPIC-Norfolk, with replications in LOLIPOP (1074 incident T2D and 1590 controls) and in cross-sectional data from FHS (403 with prevalent T2D and 2204 control participants) [29]. DNA methylation was measured in whole blood by the Illumina Infinium Human Methylation 450K BeadChip, up to 11 years before T2D onset. This study confirmed three CpG sites near to *TXNIP*, *ABCG1*, and *SREBF1*, which have been identified previously. One CpG site, cg00574958 at *CPT1A*, was found to have a possible causal role in T2D. More recently, a meta-analysis of EWASs in blood samples, collected 7–10 years prior to T2D diagnosis, was conducted by Fraszczyk et al. [39]. DNA methylation was measured with Illumina Methylation arrays among 1250 T2D cases and 1950 controls, from five prospective cohorts (450 K array for KORA, ESTHER, EPIC-Norfolk, LOLIPOP and the 850 K array for Doetinchem). A total of 76 CpG sites were identified to be associated with incident T2D, top CpG sites, including previously identified CpG sites near *TXNIP*, *ABCG1*, *SREBF1*, and *CPT1A*. After adjustment for BMI, only four CpG sites (cg19693031 at *TXNIP*, cg06500161 at *ABCG1*, cg21234053 at *CFL2*, and cg14956201 at *TRIO*) remained genome-wide significant, indicating that obesity explained much of the association between DNA methylation and incident T2D. Future studies with larger sample size are warranted to examine the DNA methylation makers predictive of T2D incidence, independent of BMI.

### 2.3. DNA Methylation and CVD

Cardiovascular diseases remain the leading cause of death worldwide and impose huge burdens on morbidity, quality of life, as well as substantial economic cost on public health systems [40,41]. It is estimated that about 659,000 individuals in the United States die from CVD every year [41,42]. Several lines of evidence from cross-sectional settings have shown that the DNA methylation profiles are different between individuals with and without CVD, suggesting a role of epigenetics in the etiopathogenesis of CVD [43,44,45,46,47,48,49]. For example, Rask-Andersen et al. conducted an EWAS study for CVD in the northern Sweden population health study, using blood samples assayed on the Illumina Infinium HumanMethylation450 BeadChip. Differential DNA methylation was observed at 211 CpG sites in individuals with a history of myocardial infarction. These sites contain genes related to cardiac function, CVD, cardiogenesis, and recovery after ischemic injury [46]. Li et al. performed a two-stage epigenome-wide methylation association analysis in two independent Chinese populations [47]. The study included 103 acute coronary syndrome (ACS) patients and 103 age-, sex-, and BMI-matched controls for the discovery stage, and another 103 ACS patients and 103 controls for the replication set. A total of 47 CpG sites were found to be reproducibly associated with ACS, with 26 CpG sites showing correlation with differential expressions in genes *IL6R*, *FASLG*, and *CCL18*. Nakarochi et al. performed an EWAS among an elderly Japanese population [48]. Epigenome-wide DNA methylation of whole blood samples of 192 myocardial infarction (MI) cases and 192 controls were profiled by the Infinium HumanMethylation450 BeadChip. The study identified three CpG sites (cg06642177, cg07786668, cg17218495) associated with MI, surpassing the genome-wide significance. Two CpG sites, located in *ZFHX3* and *SMARCA4*, remained significant, even after adjustment for traditional MI risk factors, indicating possible epigenetic mechanisms through these DNA methylation sites. In addition, a recent cross-sectional EWAS of carotid intima-media thickness (cIMT, an index for subclinical atherosclerosis) reported one CpG, cg05575921(*AHRR*), was associated with cIMT [49]. Results from the Mendelian randomization suggested a potential causal effect of cg05575921 on cIMT. Of note, these pieces of evidence were from cross-sectional studies, which limited the ability to decipher whether the disease changed the epigenetic profile or vice versa. 

In recent years, several EWASs have been conducted in prospective settings, linking DNA methylation with incident CVD (Table 1) [44,50,51,52,53,54]. For example, Westerman et al. undertook module- and region-based DNA methylation analyses of incident CVD in the Women’s Health Initiative (WHI) and Framingham Heart Study Offspring Cohort (FHS) [54]. Since previous studies linking CVD and DNA methylation, particularly in the single CpG site level, showed a notable lack of replication [54,55], the investigators aggregated the CpG sites at group level, using the weighted gene correlation network analysis (WGCNA). The methylation levels of two modules related to development and monocyte biology, and three regions associated with genes *SLC9A1*, *SLC1A5*, and *TNRC6C*, were found to be associated with CVD risk. Another study worth mentioning was conducted by Agha et al. [50]. This meta-analysis included 11,461 individuals from 9 population-based cohorts, with a mean follow-up of more than 11 years. A total of 52 CpG sites were associated with incident CHD or MI. The most protective association was found between DNA methylation at cg12766383 (*UBR4*, HR per 5% increase in DNA methylation: 0.54; 95% CI: 0.42, 0.69), while the risk increased 65% for DNA methylation at cg05820312 (*TRAPPC9*, HR per 5% increase in DNA methylation: 1.65; 95% CI: 1.35, 2.03), independent of traditional risk factors. Furthermore, Mendelian randomization analyses supported a causal relationship between DNA methylation at two CpG sites (cg26470101 and cg07289306) and incident CHD. Together, the current data suggest a possible role of DNA methylation regulation in the pathways to CVD risk.

## 3. Nutrition and DNA Methylation

Nutrition plays a pivotal role in modulating DNA methylation. The main methyl donor for DNA methylation is S-adenosylmethionine (SAM), which is a substrate derived from the one-carbon metabolism. The one-carbon metabolism is catalyzed by several enzymes in the presence of dietary micronutrients, such as folate, methionine, choline, and other B vitamins, making DNA methylation highly dependent on the availability of nutrients [56].

Nutrition status at various life stages may impact DNA methylation. The most sensitive time window for epigenetic changes in response to the environmental factors is the period of developmental plasticity, when epigenetic marks undergo critical modifications [57]. During fetal development, malnutrition of the mother may introduce the offspring to epigenetic changes in the expression of the genes associated with diseases. This was learned from the pioneering work of famine studies. Heijmans et al. reported that individuals who were prenatally exposed to famine during the Dutch Hunger Winter (1944–1945) had a lower level of DNA methylation of the imprinted insulin-like growth factor II (*IGF2*) gene 60 years later, compared to their unexposed, same-sex siblings [58]. IGF2 is an important protein involved in growth and development, particularly during the fetal stage [59]. A following study in the Dutch famine cohort also indicated that adverse prenatal nutrition could trigger widespread and persistent changes in DNA methylation, depending on the sex of the exposed individual and the gestational timing of the famine exposure [60]. Later on, the epigenome-scale analysis of differential methylation in whole blood, in the same cohort, reported that prenatal-malnutrition-associated differentially methylated regions (P-DMRs) preferentially occurred at regulatory regions. Further exploration showed that differential methylation of the P-DMRs extended along the developmental and metabolic pathways, including birth weight and lipid metabolism (DNA methylation at the intragenic *INSR* and *CPT1A*, respectively) [61]. More recently, Tobi et al. demonstrated that DNA methylation mediated the association between prenatal famine exposure with adult BMI and triglycerides [62]. Similar observations of maternal famine exposure on changes in DNA methylation in the offspring were also reported in other studies [63,64,65,66]. 

In addition to malnutrition, deficiencies of several nutrients or nutritional alterations during pregnancy have also been related to DNA methylations in the offspring [67,68,69,70,71,72]. For example, taking folic acid supplements (doses >400 μg/day) during pregnancy was associated with significantly lower levels of methylation at DNA sequence, regulating *IGF2* expression, particularly among male infants [67]. Similarly, another study found that folic acid supplementation was directly associated with the DNA methylation status of the *IGF2* gene among infants up to 17 months. Godfrey et al. conducted an analysis to examine macronutrient composition in early pregnancy and found that lower intake of carbohydrates was associated with significantly higher methylation *RXRA* gene, which was related to increased childhood body mass index and child fat mass [68].

Maternal overnutrition (such as obesity and diabetes) is also associated with DNA methylation changes. Various evidence has shown that maternal obesity is associated with increased offspring birthweight and cardiometabolic health in early childhood [73,74,75]. DNA methylation modification is hypothesized as one of the potential mechanisms. For example, data from the Newborn Epigenetics Study (NEST) cohort reported that maternal obesity was associated with several CpG sites, in or near the *TAPBP* gene in offspring. Further analyses found that pre-pregnancy maternal obesity-related CpG sites were associated with BMI z-score and blood pressure in offspring [73]. Another cross-sectional study observed that the offspring of obese mothers presented several CpG sites differentially methylated in cord blood, compared to those from normal-weight mothers [76]. An EWAS in gestational diabetes mellitus (GDM) and controls showed that exposure to GDM was associated with a group of DNA methylation variations among 9- to 16-year-old offspring. Intriguingly, the associations between GDM and differentially methylated CpG sites among offspring were confounded by maternal obesity, implicating a critical role of maternal obesity in the mechanism of epigenetic changes in the offspring of women with GDM [77]. Another study investigated the association between GDM exposure and methylation in maternal blood and offspring cord blood, among 536 mother–offspring pairs, from the prospective FinnGeDi cohort, using Illumina MethylationEPIC 850K BeadChip array [77]. The study did not observe any CpG sites with shared and consistent effects between mothers and offspring. However, after adjusting for maternal methylation in the model, one CpG site, cg22790973 (*TFCP2*), was associated with GDM at false discovery rate (FDR) 1.38 × 10^−2^. Additional FDR-significant interactions of maternal methylation and GDM was also reported, with the strongest one at the same cg22790973 (*TFCP2*), followed by cg03456133, cg24440941 (*H3C6*), cg20002843 (*LOC127841*), cg19107264, and cg11493553, located within the *UBE3C* gene, and cg17065901 in *FAM13A*, both susceptibility genes for T2D and BMI, and cg23355087 within the *DLGAP2* gene, known to be involved in insulin resistance during pregnancy. A recent study meta-analyzed eight birth cohorts, investigating relations between cord blood DNA methylation and fetal exposure to maternal glucose, insulin, and area under the curve of glucose (AUC_gluc_), following an oral glucose tolerance test [78]. The study did not find robust associations between maternal prenatal glucose and insulin levels and cord blood DNA methylation in offspring. However, greater maternal AUC_gluc_ was found to be inversely associated with cord blood DNA methylation at two neighboring CpG sites (cg26974062 and cg02988288), located within the exon of *TXNIP*. Again, stratified analyses showed these associations were attenuated among participants with GDM. A lower level of DNA methylation at these two sites was associated with several glycemic traits.

There is also evidence showing the association between maternal obesity and DNA methylation changes in several genes involved in leptin/adiponectin systems, the cytokines secreted from adipose tissue [79,80]. For example, Bouchard et al. found that placental leptin gene DNA methylation levels were associated with a 2-h post-oral glucose tolerance test (OGTT) in mothers with impaired glucose tolerance (IGT) [80]. Such results support that DNA methylation may contribute to the detrimental health effects associated with fetal programming, particularly the risk of obesity and T2D. Several studies also reported a link between other maternal exposures, such as maternal smoking, with altered DNA methylation in offspring at birth, and even during adulthood [81,82,83]. Data from the GECKO Drenthe, a Dutch-population-based birth cohort, showed that DNA methylation at the *GFI1* gene could explain 12–19% of the association between maternal smoking and low birthweight observed in the study participants [84]. Taken together, these data suggest that prenatal nutritional conditions may have a lifelong impact on shaping the offspring’s epigenome. 

After birth, when the cell or tissue is fully developed, although the plasticity of epigenetic changes is less sensitive to environmental stimuli, DNA methylation is still labile in response to nutritional status [57]. For example, one previous study has examined the relationship of alcohol and dietary folate with DNA methylation, on an epigenome-wide scale [85]. The study investigated the DNA methylation profiles of 162 well-annotated primary breast tumor samples, using the Illumina GoldenGate methylation bead-array platform [85]. The investigators found significant and independent associations between both alcohol and folate intake and overall tumor DNA methylation profiles [85]. Evidence from meta-EWAS of whole blood samples also supported that alcohol intake was associated with DNA methylation signatures [86]. A study analyzed a total of 13,317 participants of European ancestry and African ancestry and identified hundreds of differentially methylated CpG sites in relation to alcohol consumption [86]. Another study performed an EWAS meta-analysis of coffee and tea consumption among 15,789 participants of European and African-American ancestries from 15 cohorts [87]. The study revealed 11 CpG sites associated with coffee consumption, surpassing the epigenome-wide significance at *p*-value < 1.1 × 10^−7^, which annotated to genes, including *AHRR*, *F2RL3*, *FLJ43663*, *HDAC4*, *GFI1* and *PHGDH*. Among them, cg14476101 was shown to be related to *PHGDH* expression and risk of fatty liver disease. No DNA methylation marker was found for tea consumption. Another study, conducted among young women (age 20–30 years), found that moderate folate depletion might cause a decrease in global DNA methylation [88]. Following the folate depletion, women with an *MTHFR* 677 TT genotype had a better improvement in DNA methylation status, compared to women with the CC genotype [88]. Recently, Ma et al. performed a large EWAS of diet quality, assessed by Mediterranean-style Diet Score (MDS) and the Alternative Healthy Eating Index (AHEI) score, with peripheral blood-derived DNA methylation among 6662 participants of European ancestry, 2702 of African ancestry, and 360 of Hispanic ancestry, from 5 population-based cohorts [89]. A total of 30 CpG sites were identified to be associated with either MDS or AHEI, or both. Among them, hypermethylation of cg18181703 (*SOCS3*) was associated with higher scores of both MDS and AHEI, as well as lower risk for all-cause mortality. Functional analyses also demonstrated the role of many of the identified CpG sites in diet-associated pathways, involving fatty acid metabolism and insulin signaling. These findings showed that diet quality was associated with differential blood DNA methylation levels, which may reveal novel insights into the epigenetic mechanisms governed by dietary factors. Future studies with deeper coverage of DNA methylation and more precise assessment of dietary intakes are needed to validate the findings.

## 4. Diet and Lifestyle Interventions on DNA Methylation

Weight-loss diet and lifestyle interventions have been shown to have a profound effect in reducing body weight, as well as other metabolic risk factors, through changes in insulin signaling, fat storage, energy expenditure, and appetite control [90]. It has been suggested that DNA methylation changes might be one of the underlying mechanisms [91]. There is growing evidence from intervention trials showing that DNA methylation profile could be changed by diet and exercise interventions (Table 2) [80,92,93,94,95,96]. For example, Yaskolka Meir et al. conducted a study in the CENTRAL MRI randomized controlled trial, an 18-month lifestyle intervention of either low fat or Mediterranean/low carbohydrate diets [97,98]. DNA methylation age (mAge), a blood methylation biomarker for aging process, was measured among 120 participants at baseline and 18 months after lifestyle intervention, by Illumina HumanMethylation850 BeadChip [98]. The observed mAge change was significantly lower than the expected mAge change (7.1 ± 23.4 months vs. 14.8 ± 35.8 months, *p* = 0.048), among the older participants with median age of 48 years [98]. Similar to weight loss, the changes in mAge did not significantly differ across the intervention arms [98]. In addition, the study also found that the 18-month mAge change was significantly lower among the weight-loss successors (those who lost weight >5% of the initial body weight, *n* = 39) than the weight-loss failures (those who lost <5% or gained weight, *n* = 81) [98]. A similar pattern was also observed among participants with healthy liver status (intrahepatic fat <5% at the end of the study, *n* = 75), compared to participants with fatty liver (intrahepatic fat >5%, *n* = 45) [98].

**Figure 1:**
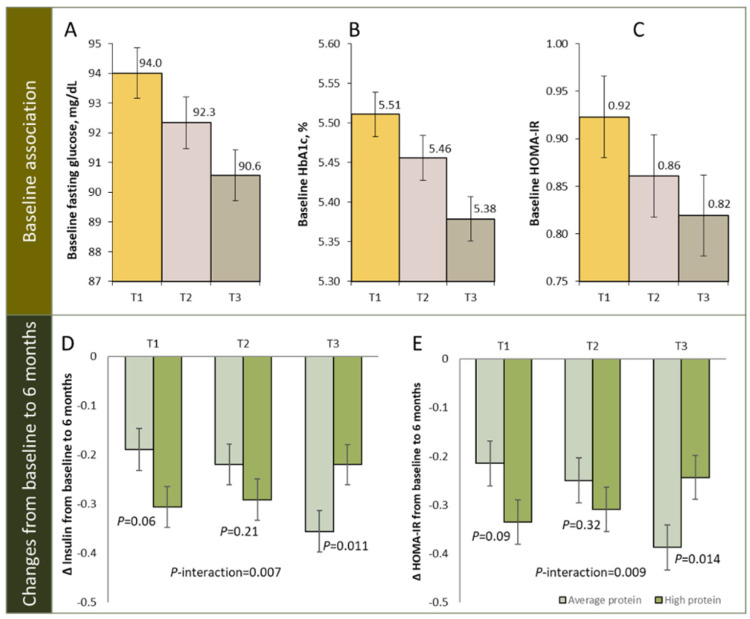
Regional DNA methylation at *TXNIP* and glycemic traits. Panel (**A**–**C**): baseline association of DNA methylation at TXNIP with fasting glucose (**A**), HbA1c (**B**), and HOMA-IR (**C**), panel (**D**,**E**): changes in insulin (**D**) and HOMA-IR (**E**) from baseline to 6 months according to tertiles of regional DNA methylation at TXNIP in average- and high-protein diet group. T1: lowest tertile; T3: highest tertile. Figure adapted from source: Li et al. *Int. J. Obes.* **2022**.

In addition, several studies have shown that pre-treatment DNA methylation profiles may serves as biomarkers that may predict individual responsiveness to weight-loss intervention (Table 2) [80,94,99,101,103]. We recently conducted a study in the POUNDS Lost trial, which is a 2-year randomized clinical trial, testing the effects of four energy-reduced diets, with varying macronutrient compositions [99,104]. A total of 639 participants with available DNA methylation data at baseline were included and 516 participants had completed the trial at 2 years. We calculated the regional DNA methylation at *TXNIP* as the average methylation level over CpG sites, within 500 bp of cg19693031, one of the most robust sites associated with insulin resistance [105], islet dysfunction [106,107], hyperglycemia [27], and T2D [27,29,38]. We found that higher baseline regional DNA methylation levels at *TXNIP* were associated with lower levels of fasting glucose (*p* < 0.001), insulin (*p* < 0.001), and homeostatic model assessment for insulin resistance (HOMA-IR, *p* = 0.03) (Figure 1). In addition, we observed that dietary protein significantly modified the association between regional DNA methylation at *TXNIP* and 6-month changes in insulin and HOMA-IR (*p*-interaction = 0.007 and 0.009, respectively). Among participants with the highest tertile of regional DNA methylation at *TXNIP*, average protein intake (15%) was associated with greater reduction in insulin and HOMA-IR compared to high protein intake (25%), while no association was found among those within the lower tertiles. Of note, such relationship was independent of concurrent weight-loss. The interaction pattern was attenuated and became non-significant at 2 years, presumably due to the decreased adherence to the diet interventions [99]. This data, together with others listed in Table 2, suggest that DNA methylation could be used as a predictive biomarker for weight-loss outcomes. 

In addition, although in small sample sizes, some, but not all, of the studies found that after gastric bypass surgery, the DNA methylation profiles of individuals with obesity were similar to those of normal-weight individuals [100,101,102,108,109]. Collectively, these results indicate that DNA methylation could be changed by weight-loss diets and other lifestyle interventions.

## 5. Other Epigenetic Events

Besides DNA methylation, other epigenetic events, such as non-coding RNAs, are also involved in the pathogenesis of obesity, T2D, and CVD (Table 3). Non-coding RNAs are functional RNAs that are transcribed from DNA but not translated into proteins. Currently, miRNA and lncRNA are found to be the most functionally relevant to obesity. MiRNAs are a group of small RNAs (approximately 22 nucleotides) that post-transcriptionally regulate gene expression. Over the past two decades, miRNA has been increasingly recognized as important regulators of biological process linked to T2D and various cardiovascular pathologies, such as left ventricular hypertrophy, myocardial infarction (MI), atherosclerosis, heart failure, and arrhythmias [110,111,112]. MiR-21 appeared to be one of the top hits associated with CVD and obesity [113,114]. Mounting evidence has linked miR-21 with many cardiovascular diseases [115,116,117]. Functional analyses found that miR-21 played a critical role in these cardiovascular disorders [116]. A previous study, conducted among 501 MI patients and 87 healthy controls, found that MI was associated with a substantial deregulation of circulating miRNAs and lncRNAs [118]. As such, miR-208b and miR-499 were substantially increased in MI patients (>10^5^-fold), whereas no elevation was detected among healthy individuals. Further, miR-499 also showed comparable diagnostic values with high-sensitivity cardiac troponin T (hs-cTnT), with areas under the receiver operating characteristics curves of 0.97. In addition to miRNA, the role of long non-coding RNA, in the diagnosis, prognosis, and clinical management of obesity, T2D, and CVD, has also been evidenced in growing studies [119,120,121,122,123,124,125]. Recent studies have uncovered thousands of lncRNA in human pancreatic β cells and islets [126,127,128].

Emerging evidence has shown that diet may alter the miRNA profiles [139,140,141,142]. For example, Wang et al. investigated the association between changes in circulating miR-122 and liver fat, in response to weight-loss diet interventions [140]. After 18-month diet and physical activity interventions, serum miR-122 significantly increased. In addition, greater elevations in miR-122 were associated with fewer reductions in liver fat percentage. Another study, conducted by Assmann et al., reported several miRNAs were differentially expressed between responders and non-responders to a low-fat diet, indicating that miRNA could be served as early predictors of weight loss in precision nutrition [141]. Though the data are still scarce, these previous studies lend support to the potentially important role of various epigenetic events in determining individual variability in health status and response to diets.

## 6. Summary and Future Directions

In summary, current data support the view that epigenetic events, particularly DNA methylation, might play an important role in the pathogenesis of obesity, T2D, and cardiovascular diseases. Although there are few overlaps of DNA methylation markers in previous studies, compared to the number of single nucleotide polymorphisms identified in genomic studies, the current evidence collectively suggest that DNA methylation is associated with cardiometabolic disorders. The growing evidence has also shown that DNA methylation is closely related to nutrition status at various life stages, including fetal development, early life, and adulthood. Considering the dynamic and modifiable nature of DNA methylation, it could be a target for future precision dietary interventions in the prevention and treatment of cardiometabolic diseases. Emerging data have lent support to the potential effectiveness of diet and lifestyle interventions on changes in epigenetic events, especially DNA methylation and miRNAs. Once validated, these identified epigenetic markers could serve as novel targets for dietary and lifestyle interventions. More future studies, with larger sample size, prospective design, high-coverage and repeated measurements on DNA methylations and other epigenetic events, are still warranted. Of note, since epigenetic events are cell specific, it is essential to investigate the tissue-specific epigenetic modifications in future studies. It is also important to examine more complex interactions of epigenetic markers and dietary factors with other lifestyle factors, such as physical activity, sleep behaviors, and other environmental factors, in determining the individual variability in relation to precision health. 

## Figures and Tables

**Table 1:** Selected DNA methylation loci for obesity, T2D, and CVD.

Disease	CpG Site	Gene	Gene Summary *
Obesity	cg00574958 †, cg09737197 †, cg17058475 †, cg01082498 †	*CPT1A*	*CPT1A* (Carnitine Palmitoyltransferase 1A) is a Protein Coding gene. Diseases associated with *CPT1A* include Carnitine Palmitoyltransferase I Deficiency and Carnitine Palmitoyltransferase Ii Deficiency, Infantile. Among its related pathways are Import of palmitoyl-CoA into the mitochondrial matrix and Regulation of lipid metabolism by Peroxisome proliferator-activated receptor alpha. Gene Ontology (GO) annotations related to this gene include identical protein binding and carnitine O-palmitoyltransferase activity.
	cg06500161	*ABCG1*	*ABCG1* (ATP Binding Cassette Subfamily G Member 1) is a Protein Coding gene. Diseases associated with *ABCG1* include Tangier Disease and Sitosterolemia. Among its related pathways are Nuclear Receptors in Lipid Metabolism and Toxicity and Lipoprotein metabolism. GO annotations related to this gene include protein homodimerization activity and GTP binding.
	cg11024682 †	*SREBF1*	*SREBF1* (Sterol Regulatory Element Binding Transcription Factor 1) is a Protein Coding gene. Diseases associated with *SREBF1* include Ifap Syndrome 2 and Mucoepithelial Dysplasia, Hereditary. Among its related pathways are ID signaling pathway and SREBF and miR33 in cholesterol and lipid homeostasis. GO annotations related to this gene include DNA-binding transcription factor activity and chromatin binding.
	cg22891070, cg27146050, cg16672562	*HIF3A*	*HIF3A* (Hypoxia Inducible Factor 3 Subunit Alpha) is a Protein Coding gene. Diseases associated with HIF3A include Hypoxia. Among its related pathways are Hypoxic and oxygen homeostasis regulation of HIF-1-alpha and CDK-mediated phosphorylation and removal of Cdc6. GO annotations related to this gene include DNA-binding transcription factor activity and transcription coactivator activity.
T2D	cg19693031	*TXNIP*	*TXNIP* (Thioredoxin Interacting Protein) is a Protein Coding gene. Diseases associated with *TXNIP* include Leukostasis and Hyperglycemia. Among its related pathways are Nucleotide-binding domain, leucine rich repeat containing receptor signaling pathways and Innate Immune System. GO annotations related to this gene include ubiquitin protein ligase binding and enzyme inhibitor activity.
	cg06500161	*ABCG1*	*ABCG1* (ATP Binding Cassette Subfamily G Member 1) is a Protein Coding gene. Diseases associated with *ABCG1* include Tangier Disease and Sitosterolemia. Among its related pathways are Nuclear Receptors in Lipid Metabolism and Toxicity and Lipoprotein metabolism. GO annotations related to this gene include protein homodimerization activity and GTP binding.
	cg11024682	*SREBF1*	*SREBF1* (Sterol Regulatory Element Binding Transcription Factor 1) is a Protein Coding gene. Diseases associated with *SREBF1* include Ifap Syndrome 2 and Mucoepithelial Dysplasia, Hereditary. Among its related pathways are ID signaling pathway and SREBF and miR33 in cholesterol and lipid homeostasis. GO annotations related to this gene include DNA-binding transcription factor activity and chromatin binding.
	cg00574958 †	*CPT1A*	*CPT1A* (Carnitine Palmitoyltransferase 1A) is a Protein Coding gene. Diseases associated with *CPT1A* include Carnitine Palmitoyltransferase I Deficiency and Carnitine Palmitoyltransferase Ii Deficiency, Infantile. Among its related pathways are Import of palmitoyl-CoA into the mitochondrial matrix and Regulation of lipid metabolism by Peroxisome proliferator-activated receptor alpha. GO annotations related to this gene include identical protein binding and carnitine O-palmitoyltransferase activity.
CVD	cg12766383	*UBR4*	*UBR4* (Ubiquitin Protein Ligase E3 Component N-Recognin 4) is a Protein Coding gene. Diseases associated with UBR4 include Retinoblastoma and Episodic Ataxia. Among its related pathways are Innate Immune System and Class I MHC mediated antigen processing and presentation. GO annotations related to this gene include binding and ubiquitin-protein transferase activity.
	cg05820312	*TRAPPC9*	*TRAPPC9* (Trafficking Protein Particle Complex Subunit 9) is a Protein Coding gene. Diseases associated with *TRAPPC9* include Intellectual Disability-Obesity-Brain Malformations-Facial Dysmorphism Syndrome and Autosomal Recessive Non-Syndromic Intellectual Disability. Among its related pathways are Transport to the Golgi and subsequent modification and Vesicle-mediated transport.
	cg26470101 †	*DLX2*	*DLX2* (Distal-Less Homeobox 2) is a Protein Coding gene. Diseases associated with *DLX2* include Axenfeld-Rieger Syndrome and Split-Hand/Foot Malformation 5. Among its related pathways are Sudden Infant Death Syndrome. Susceptibility Pathways and Preimplantation Embryo. GO annotations related to this gene include DNA-binding transcription factor activity and chromatin binding.
	cg07289306 †	MIR138−1	MIR138-1 (MicroRNA 138-1) is an RNA Gene, and is affiliated with the miRNA class. Diseases associated with MIR138-1 include Oral Squamous Cell Carcinoma and Thyroid Cancer, Nonmedullary, 1.

* Quoted from the GeneCards^®^: The Human Gene Database http://www.genecards.org/. (Accessed date: 26 February 2022) † Denotes potential causal association.

**Table 2:** DNA methylation in weight-loss diet and lifestyle interventions.

Intervention	Population	Tissue	Methylation Sites/Method	Main Findings
2-year diet interventions (4 energy-reduced diets with varying macronutrient compositions) [99]	639 overweight/obese participants with available DNA methylation at baseline	Blood	Genome-wide/high-resolution methylC-capture sequencing	Among average-protein group, higher regional DNA methylation levels at *TXNIP* (average methylation level over CpG sites within 500 bp of cg19693031) was associated with greater reduction in insulin and HOMA-IR at 6 months
18 months lifestyle intervention including diet and physical activity (Mediterranean low-carb (MED/LC) vs. low-fat (LF) vs. MED/LC + physical activity vs. LF + physical activity) [92]	A total of 120 sedentary adults with abdominal obesity or dyslipidemia (110 male and 10 female)	Blood	Genome-wide/Illumina HumanMethylation850 BeadChip	Differences were observed in 8 differentially methylated regions (DMRs) around 9 genes between 10 responders (mean weight change −16%) and 10 non-responders (+2.4%), including *LRRC27*, *CRIPSP2*, *SLFN12*, *LINC00539*, *AURKC*, *RNF39*, *RP11-283I3.2*, *SLC6A12*, *NTSR1*. Among all the 120 participants, 47 CpG sites (15 CpG sites correlated negatively and 32 positively) showed significant correlation with weight loss after intervention.
18 months lifestyle intervention including diet and physical activity (Mediterranean low-carb (MED/LC) vs. low-fat (LF) vs. MED/LC + physical activity vs. LF + physical activity) [98]	A total of 120 sedentary adults with abdominal obesity or dyslipidemia (110 male and 10 female)	Blood	Genome-wide/Illumina HumanMethylation850 BeadChip	Lifestyle intervention attenuated DNA methylation age (mAge) of the men, particularly among the older participants. No difference between the mAging between the intervention groups. The 18-month changes in mAge among 39 weight-loss successors was significantly lower than that of the 81 weight-loss failures. Similarly, participants with healthy liver fat % (intrahepatic fat <5%, *n* = 75) at the end of the intervention had significantly lower mAge, compared to those participants with fatty liver (*n* = 45, intrahepatic fat >5% at the end of the intervention).
6 months exercise intervention [93]	A total of 23 healthy overweight men	Adipose tissue	Genome-wide/Illumina HumanMethylation450 BeadChip	Global DNA methylation changed after exercise intervention. A total of 17,975 individual CpG sites in 7663 unique genes showed altered levels of DNA methylation. Also, 18 obesity and 21 T2D candidate genes had CpG sites with differences in adipose tissue DNA methylation, including *TCF7L2* (6 CpG sites) and *KCNQ1* (10 CpG sites).
10 weeks multidisciplinary intensive lifestyle intervention [94]	First methylation array was conducted among 24 obese or overweight adolescents (12 high responders and 12 low responders); second, a validation analyses was performed in 107 adolescents	Blood	Genome-wide/Illumina Infinium HumanMethylation27 BeadChip27k, validation using Sequenom EpiTyper MassARRAY followed by MALDI-TOF mass spectrometry	Comparing the baseline differences between high responders and low responders revealed 97 CpG sites with >5% changes in DNA methylation, validation analysis showed 5 regions with differential methylation levels, including *AQP9*, *DUSP22*, *NIPK3*, TNNT1, and *TNNI3*.
6 months caloric restriction intervention [80]	Overweight/obese postmenopausal women (7 high responders and 7 low responders)	Subcutaneous adipose tissue	Genome-wide/Human CpG-island 8.1 K array and 6800 additional CpG island loci, validation by Sequenom EpiTyper MassARRAY	At baseline, significant DNA methylation differences at 35 loci were found between the high and low responders before intervention. After intervention, 3 regions showed differential methylation. Some of the identified regions contains genes known to be related to weight control and insulin secretion, or in known imprinted genomic regions.
6 months exercise intervention [95]	15 men with (F+) and 13 men without (F-) a first-degree family history of T2D	Skeletal muscle	Genome-wide/Infinium HumanMethylation450 BeadChip, validation by Sequenom EpiTyper MassARRAY	A total of 134 individuals genes changed in DNA methylation level after intervention. The identified genes include those in the retinol metabolism and calcium signaling pathways and with known functions in muscle and T2D, such as *MEF2A*, *RUNX1*, *NDUFC2*, and *THADA*.
1-year weight loss intervention (weight loss diet and exercise) [96]	19 healthy obese participants	Subcutaneous adipose tissue	Genome-wide/Infinium HumanMethylation450 BeadChip	No genome-wide significant differentially methylated CpG sites were observed (baseline vs. 5 months, 5 months vs. 12 months, or baseline vs. 12 months)
RYGB [100]	Obese women with RYGB surgery (*n* = 8) and nonobese (normal weight) glucose-tolerant age-matched women (*n* = 9); obese men before and after RYGB surgery (*n* = 6)	Skeletal muscle	*PGC1α* and *PDK4* promoter region and 14 other genes by bisulfite sequencing among women; Genome-wide methylation analysis for men by bisulfite sequencing	RYGB surgery decreased *PGC-1α* promoter methylation and conversely increased *PDK4* promoter methylation. Among the 14 metabolic genes analyzed, promoter methylation of 11 genes was normalized to levels in normal weight individuals. In men, 409 DMRs were found pre- and post-RYGB.
Energy restriction diet and bariatric surgery [101]	Obese women with energy restriction diet (*n* = 22); obese women underwent a hypocaloric dietary treatment followed by RYGB (*n* = 14); normal weight women (*n* = 9)	Blood	Methylation at *LINE-1*, *IL-6*, and *SERPINE-1* accessed by Methylation-Sensitive High-Resolution Melting (MS-HRM)	*LINE-1* and *SERPINE-1* methylation levels did not change after weight loss (energy restriction and RYBG), *IL-6* methylation increased after energy restriction and decreased in the bariatric surgery group. Baseline *SERPINE-1* methylation levels were significantly lower in the high responders to the RYBG.
Bariatric surgery [102]	45 obese patients with NAFLD; 23 participants underwent bariatric surgery	Liver	Genome-wide/Illumina HumanMethylation450 BeadChip	Post-bariatric and NAFLD-specific methylation signatures were clearly distinct. The gene encoding protein-tyrosine phosphatase epsilon (*PTPRE*) showed a differential methylation before and after bariatric surgery.

**Table 3:** Selected promising miRNAs associated with obesity, T2D, and CVD.

MicroRNA ID	Changes in Expression	Associated Disease/Conditions	Ref.
miR-221	↑	obesity/subcutaneous adipose tissue	[129,130]
miR-128-1	↑	thrifty phenotype (high glucose, insulin resistance, energy storage, ets.)	[131]
miR-103/107	↑	obesity, alcoholic liver disease (ALD), non-alcoholic fatty liver disease (NAFLD) and non-alcoholic steatohepatitis (NASH)	[132]
miR-140-5p, miR-142-3p, miR-222	↑	T2D	[133]
miR-423-5p, miR-125b, miR-192, miR-195, miR-130b, miR-532-5p, miR-126	↓		
miR-21	↑	proliferative vascular disease, cardiac hypertrophy and heart failure, and ischemic heart disease.	[113,114,117]
miR-1254	↑	chronic heart failure	[134]
miR-30d	↓	heart failure	[135]
miR-1306	↑	acute heart failure	[136]
miR-1, miR-133a, miR-133b, miR-499-5p	↑	myocardial infarction	[137]
miR-122, miR-375	↓		
miR-223, miR-328, miR-664	↑	atrial fibrillation	[138]
miR-101, miR-320, miR-499	↓		

↑ denotes increased expression and ↓ indicates decreased expression in miRNA.

## Data Availability

Not applicable.
